# Tactile massage and hypnosis as a health promotion for nurses in emergency care-a qualitative study

**DOI:** 10.1186/1472-6882-11-83

**Published:** 2011-10-01

**Authors:** Fanny Airosa, Susanne K Andersson, Torkel Falkenberg, Christina Forsberg, Elisabeth Nordby-Hörnell, Gunnar Öhlén, Tobias Sundberg

**Affiliations:** 1 Unit for Studies of Integrative Health care-Karolinska Institutet, Department of Neurobiology, Care Sciences and Society, Division of Nursing, 141 83 Huddinge, Sweden; 2 Karolinska University Hospital, Emergency Department, 141 86 Stockholm, Sweden; 3 The Vidarinstitutet Research Foundation, 153 91 Järna, Sweden

**Keywords:** emergency care nursing personnel, health promotion, qualitative content analysis, experience, perceptions, complementary therapies, tactile massage, hypnosis

## Abstract

**Background:**

This study explores nursing personnel's experiences and perceptions of receiving tactile massage and hypnosis during a personnel health promotion project. Nursing in a short term emergency ward environment can be emotionally and physically exhausting due to the stressful work environment and the high dependency patient care. A health promotion project integrating tactile massage and hypnosis with conventional physical activities was therefore introduced for nursing personnel working in this setting at a large university hospital in Sweden.

**Methods:**

Four semi-structured focus group discussions were conducted with volunteer nursing personnel participants after the health promotion project had been completed. There were 16 participants in the focus groups and there were 57 in the health promotion intervention. The discussions were transcribed verbatim and analysed with qualitative content analysis.

**Results:**

The findings indicated that tactile massage and hypnosis may contribute to reduced levels of stress and pain and increase work ability for some nursing personnel. The sense of well-being obtained in relation to health promotion intervention with tactile massage and hypnosis seemed to have positive implications for both work and leisure. Self-awareness, contentment and self-control may be contributing factors related to engaging in tactile massage and hypnosis that might help nursing personnel understand their patients and colleagues and helped them deal with difficult situations that occurred during their working hours.

**Conclusion:**

The findings indicate that the integration of tactile massage and hypnosis in personnel health promotion may be valuable stress management options in addition to conventional physical activities.

## Background

To work as a nurse in a short term emergency ward can be emotionally and physically exhausting due to its stressful environment [1]. One aspect of work-related stress is the lack of resources to meet constant interruptions (e.g. telephones and the cluttered work environment) [2]. There are many additional factors that may contribute to work-related stress. One of which is inexperienced personnel as opposed to experienced personnel. For example if there is a large proportion of newly qualified or inexperienced nurses on the ward this may lead to insatisfactory work relations as their skills and competency may not meet up with the working demands and, therefore, the workload might be perceived as too high and stressful [3]. Similarly there are a variety of stress factors that have been identified which increase the risk of burnout and distress in nursing personnel. Such factors include emotional distress in contact with the severely ill. Patients close to death as well as high dependency patient care can also contribute to stress. Work related conflicts amongst the personnel are also a contributing factor to stress [4]. Stress can have a detrimental effect on the nurses' work performance which can later lead to health problems [5]. Previous research indicates that long term stress may lead to sick leave or personnel leaving the work place [6]. Over the last number of years health promotion activities have been an issue discussed widely in the Swedish Health system [7]. There are many different health promotion activities for the personnel at the university hospital, such as physical exercise classes, gym activities, biking, and stop-smoking and weight loss courses. There is also a focus on well-being in the form of choir singing, yoga and meditation [8]. An integrative care unit was designed at the end of 2004 to implement a health promotion intervention project for the nursing personnel at the short term emergency department. The complementary therapies (CTs) that were introduced at the unit were initially hypnotherapy and tactile massage therapy. CTs have been the topic of increased public interest and used in recent years for a number of reasons and one known use is to alleviate the effects of stress [9]. CT treatments may be defined as treatments that are provided largely outside the conventional or publicly funded health care system. Many CT providers have a holistic view of human illness but this holistic approach is not unique to complementary practice [10]. Earlier surveys have found increasing use of CT therapies worldwide. In the United States, use of CT therapies increased from 33.8% in 1990 to 42.1% in 1997 [11]. Consequently in a study of 174 emergency patients, 47% of those reported used also CTs [12]. In Sweden, 49% of the population in the Stockholm County reported visiting a CT provider in the year 2000, compared to 20% in the 1980s [13].

*A hypnotherapy *session entails hypnotic induction, trance-deepening instructions and imagery therapy, hypnotic suggestions are designed to produce an overall physical relaxation [14]. Experiencing a state of trance is something natural, and can even occur spontaneously such as driving on a familiar road every day or when in a boring situation [15]. Previous studies of hypnotherapy have shown to alleviate anxiety, relieve stress, and strengthen self-confidence and self-esteem [16]. The hypnotherapy given in this study was based on Milton Erickson's principles and theories of non-authoritarian and non-directional hypnosis [17].

*Massage therapy *is the most common and most utilized type of CT in Sweden [18]. It uses touch and manual techniques, such as stroking and gentle pressure, in order to relax the body and help to restore health [19]. In this study tactile massage was used, and this soft tissue massage was developed by the Swedish nurse Siv Ardeby [19]. A study on massage therapy and occupational stress on hospital personnel illustrates that massage may reduce anxiety and lead to a significant reduction in blood pressure [20].

## Aim

To explore nursing personnel's experiences and perceptions of receiving tactile massage or hypnosis as a part of personnel health promotions activities at a short term emergency ward.

## Methods

### Study design and setting

This qualitative study was conducted parallel to a nursing health promotion project to reduce work related stress for nursing personnel working at a short term emergency ward at a large University Hospital in Sweden.

### Participants

Fifty-seven nursing personnel (i.e. nurses and assistant nurses) working at the short term emergency ward volunteered to participate in the personnel health promotion project. Thirty-eight of these received the health promotion intervention with either tactile massage or hypnosis. Subsequently, sixteen of the 38 participants that had received either tactile massage or hypnosis volunteered to share their experiences and perceptions in a focus group discussion. A convenience self-selected sample was used.

### Interventions

Two nurses in the department were certified providers of tactile massage and hypnosis respectively. All health promotion interventions were provided at an integrative care unit adjacent to the short term emergency ward, and health promotion interventions were scheduled during working hours. The health promotion interventions were delivered once a week for a maximum number of eight 45-minute sessions.

#### Tactile massage

The tactile massage was performed in a quiet room with the participant lying on a massage bench, embedded in towels and blankets; the only part of the participant's body that was being touched, was uncovered. Tactile massage was given on the back, hands and feet using a neutral vegetable oil. The tactile massage was carried out with slow strokes, light pressure and circling movements, mainly done with the palm of the hand with the fingers close together [19].

#### Hypnosis

The hypnotherapy session took place in a quiet room with the participant sitting in a comfortable chair. The hypnotic process began with images of a favourite calming and relaxing image for the participant. The hypnotist used positive words (i.e., relaxation, calm, warmth and relaxing) and avoided negative words (such as pain). The hypnotist deepened the trance by instructing the participant to relax more by taking deep breaths. During the session the hypnotist repeated suggestions that induced relaxation. The session ended with the hypnotist making a count down from five to one, slowly returning the participant from the hypnotic state to present time and place [17].

### Data collection

Four focus group discussions were conducted from May 2005 to February 2006. This type of qualitative inquiry has been frequently used and is a recommended approach to explore patients' experiences and perceptions in different contexts and settings [21]. An external moderator and an assistant not involved in the clinical health promotion interventions conducted the focus group discussions which were scheduled for one hour, digitally audio-taped and transcribed verbatim. An interview guide (appendix 1) was used to guide the discussions.

### Analysis

Manifest content analysis was used to analyse the data from the focus groups [22]. The analysis was conducted in five repetitive stages: (1) reading of each interview with an intention of understanding the general content, (2) dividing the text first into meaning units, then condensed meaning unit, which were abstracted into codes (3) the codes were compared based on their differences and similarities and sorted into eleven sub-categories, (4) The sub-categories were then scrutinised and compared until five categories had emerged (5). Finally an overall theme was formulated based on interpretations of the codes, sub-categories and categories (Tables 1 and 2). Validation of all steps has been considered carefully. Two authors (FA and SA) independently performed a first reading and then discussed the content of the text together with CF. Subsequently; the text was divided by FA into meaning units, that is to say coherent portions of the content, consisting of words and/or phrases carrying an important meaning to the study. The condensed meaning units were sorted into codes and summarised into sub-categories, categories and an overall theme. To ensure interpretation and validity the derived codes, sub-categories and categories were scrutinized by FA, SA and CF, individually and together, in relation to the original texts and subsequently discussed with all co-authors until a consensus was reached.

**Table 1:** Example of meaning unit, condensed meaning, code, sub-category and category.

Meaning unit	Condensed meaning	Code	Sub-category	Category
-This project provides us with the ability to do our job, stress is a big factor in our work, and if we can manage to deal with our stress before it actually becomes an issue we can do a better job.	The project provided an ability to do our job, stress is a big factor and if we can deal with it we can do a better job	Reducing stress gives an increased ability to deal with workload	Help with stress issues	Dealing with workload

**Table 2:** Theme, categories and sub-categories

**Theme; **tactile massage and hypnosis may reduce stress and pain and increase contentment, self-awareness and work ability for some nursing personnel.					
**Categories**	I) Feeling relaxed and gaining more energy and work ability	II) Dealing with workload	III) Relieving physical and psychological pain	IV) Being treated	V) Knowing oneself
**Sub-Categories**	*Your own well-being* *affects work and* *leisure time*.	*Increased work ability*. *Help with stress issues*	*Give pain relief*	*Positive response to* *treatments connected* *to the workplace*.	*Affects self-awareness*. *Increased self-control*
	*Relaxation creates a* *feeling of harmony* *and tranquility*.			*Need for health promotions*	

	*More energy*.				

	*It is positive to be cared for*.				

### Ethical considerations

The research project was approved by the regional ethics committee Dnr 2005/831-31. Participation was voluntary and involved informed consent.

## Results

This study explores the experience and perceptions of receiving tactile massage or hypnosis for the emergency care personnel in a health promotion project. The findings indicate that tactile massage and hypnosis may help to support nursing personnel working in the short term emergency ward in dealing with a very stressful environment. The theme "Tactile massage and hypnosis may reduce stress and pain and increase well-being, self-awareness, and work ability for the nursing personnel at a short term emergency ward" including categories and sub-categories derived from the data analysis (Table 2). Five categories and eleven sub-categories formed the basis for the theme. The categories illustrated the need of a health promotion program in a physically and psychologically difficult environment as illustrated in the short term emergency ward to reduce work related stress and increase complacency and energy for its personnel.

### Category I: Feeling relaxed and gaining more energy and work ability

This category derived from the sub-categories described below and illustrates how CTs such as tactile massage and hypnosis affected the participants feeling of well-being, of being able to relax and being taken care of. The participants described their work as being physically and psychologically tiring and stressful due to difficult shifts; they had no energy or ability to socialize during their time off.

-We were very busy in the short term emergency ward for the last duration, a lot of admissions and complicated cases left us exhausted. (FG4)

#### Your own well-being affects work and leisure time

CTs such as tactile massage and hypnosis illustrate the impact of the contentment from the participants. Some participants describe how the impact of contentment affected both their life at work and in the home environment in a very positive way. Their feeling of well-being at work gave them the ability to support their colleagues and deal with stressful situations. Participants could give support to their patients and colleagues during the period they received

the health promotion intervention.

-Your approach and attitude is better towards your colleagues and patients, and the nursing care improved. (FG3)

#### Relaxation creates a feeling of harmony and tranquility

After the health promotion intervention with either hypnosis or tactile massage the participants experienced a feeling of harmony and contentment in body and soul.

- I feel very good. I feel relaxed and harmonious, and much better. (FG3)

-The weight is lifted from my shoulders; massage gives me a new lease of life like a newborn baby. (FG2)

#### More energy

Several of the participants related that they had more energy after receiving a health promotion intervention and more energy to enjoy their social life.

-You are calmer and have more energy to enjoy your time off instead of going home and lying on the sofa, no more feelings of exhaustion. (FG 3)

#### It is positive to be cared for

"It is positive to be cared for" is a sub-category which illustrates the great need to be taken care of for the nursing personnel. The participants related that by practicing nursing care, you give a lot of yourself to your patients. During the study the nurses and assistant nurses' had the opportunity to be taken care of; in either body (tactile massage) or mind (hypnosis).

-The body needed this contact (massage) the smell of lavender and the music; it felt like I needed it. (FG 2)

Another participant said that it was difficult to explain the feeling.

-I could not describe the feeling, but that is just nice that someone took care of **me**, it felt so nice afterwards. (FG 2)

Hypnosis clears your mind of all thoughts and leaves you in a place of tranquility. Even if you are feeling tired, hypnotherapy lets your brain rest as one participant described;

-In the hypnotherapy I felt the desire to be there, you want to be there and just relax "get away" for a while and just make a journey on your own. (FG 2)

### Category II: Dealing with workload

The category, dealing with workload comprised of experience and awareness relating to *increased work ability *and *help with stress issues*.

#### Increased work ability

The participants expressed an increase in their work ability and energy during the time of a health promotion intervention.

- *You calm down and get a new lease of energy when you get back to work. (FG1)*

#### Help with stress issues

Some of the participants mentioned the importance of receiving help with stress issues. Many of the participants expressed a feeling of work related stress, and said that the health promotion intervention helped them to do a better job despite the stress. Perhaps it could help the nursing personnel to prevent stress before it becomes an issue.

-This project provides us with the ability to do our job, stress is a big factor in our work, and if we can manage to deal with our stress before it actually becomes an issue we can do a better job. (FG 3)

-you don't feel stressed; you are calmer with your colleagues and patients. You learn how to deal with stress; you treat your patients and colleagues around you better when you are in a better mood. (FG 3)

### Category III: Reliving physical and psychological pain

#### Give pain relief

This category emerged from the experience of pain relief, in both physical and psychological pain. Tactile massage seemed to relieve pain for some of the participants especially for those experiencing pain such as back-pain and headaches. The health promotion interventions also seemed to give relieve in psychological pain associated with life-threatening situations.

I felt a tremendous relief during the time I received massage therapy for my back pain; it's not fully better but much improved, good enough, so I can cope with it. This was indeed a pleasant surprise. My stress related headache had also disappeared, my body felt considerable better. (FG 1)

I could not have dealt with this difficult time in my life without tactile massage treatment. I received bad news in November, and have since had a mastectomy for breast cancer. During this awful time in my life I received tactile massage treatment and felt an enormous relief afterwards, the therapist with her hands played a big part in my healing; it was her and the therapy that got me through this difficult stage in my life. (FG4)

### Category IV: Being treated

This category derived from the two sub-categories *positive response to treatments connected to the workplace *and *need for health promotion*. Participants experienced the need for these particular kinds of health promotion intervention to help them deal with the stressful working conditions and situations that could arise during a shift; there seems to be a need for work-based health promotion activities close to the work place.

#### Positive response to treatments connected to the workplace

If the therapies are located close to the working area it is more likely that the personnel would take part in the health promotion interventions. In this project it was possible for the personnel to take health promotion interventions during their shift which the participants revealed were very much appreciated by everyone at the short term emergency ward.

-To have a treatment during your shift was exhilarating, otherwise we would never have the time for it, and it was fantastic. Very much appreciated by all. (FG 1)

-You learn how to relax, the fact that you could have it during working hours! Well, I think this was and is tremendous. (FG 3)

#### Need of health promotion

There seemed to be a need for continuity in the health promotion intervention, some participants who received hypnosis felt they needed repeated health promotion interventions to learn how to relax.

-In my opinion, you should have a longer period in the beginning followed by treatments on a regular basis. (FG 3)

Body and mind therapy was a good complement to other physical activities. As one participant related;

-I use to do a lot of physical activities, but now I really feel the need for a massage therapy. (FG 2)

### Category V: Knowing oneself

This category has two sub-categories *Affects self-awareness *and *Self-control*.

#### Affects self-awareness

Many of the participants describe that their experiences in deep relaxation affected their way of dealing and treating patients and colleagues. Their tolerance and patience had reached a much higher threshold in relation to their patients and colleges than before.

-In the beginning of the study I felt that I could be very irritated and had difficulty in nursing patients who felt angry, difficult or frustrated, but I managed to change my attitude, thanks to hypnotherapy. (FG 3)

-If you are calmer and feel that you have more energy it's much easier to understand why somebody acts in a certain way and deal with it in a better way. (FG 3)

#### Self-control

To gain self-control was mentioned by some participants as a positive outcome from having hypnosis. However, one of the participants revealed, an entirely different opinion in regards to hypnosis that in losing one's self-control during hypnosis was frightening.

-You don't feel stressed as quickly when you have had a treatment. It is easier to keep everything under control. (FG 1)

-My experience with hypnotherapy; It was like losing control over my body. You just disappear. It was so frightening. Well I did not do it so often because of this experience. (FG 3)

Some of the participants pointed out that hypnosis might not suit everyone.

-I have presumptions about hypnosis, not prejudices, well you are a bit frightened of what's going to happen...what will I do...will I cry...you know, it's a bit sensitive, before this I had a massage treatment to relieve some pain in my shoulders. (FG4)

- When I talked to my colleagues the majority of them said that they liked the massage treatment but with hypnosis it was too scary because of the fear of losing control. (FG4)

Some of the participants use the regular health promotion activities at the hospital (i.e. exercise classes or gym activities) which are voluntary. There is a possibility for the personnel to shorten their working hours by one hour per week in order to perform health promotion activities in the gym, or group activities i.e. going for walks in groups around the hospital area which is in close proximity to the countryside.

The participants related that the health promotion interventions in tactile massage and hypnosis "worked" at different levels as opposed to other physical activities. Both tactile massage and hypnosis seem to be a way to clear your mind and give you a sense of tranquility. Even if the body is tired, the health promotion interventions let your mind rest which gives you a new lease of life.

## Discussion

This study explored the experiences and perceptions of nursing personnel that had received tactile massage or hypnosis during a nursing personnel health promotion intervention project at a short term emergency ward in Sweden. The findings indicate that tactile massage and hypnosis may help to support some nursing personnel in dealing with the extremely high dependency working environment. Earlier research found that emergency nurses are subjected to significant stressors during their shift, therefore a higher threshold of stress is required to deal with the high dependency and nursing care demands [1,23]. In our study the findings point out that even if the university hospital provides the opportunity to participate in physical health promotion activities there is also a need for relaxation to allow the mind and body to rest. A previous study indicate that interventions such as cognitive-behavioral therapy and relaxation techniques may be effective in reducing stress, anxiety, and burnout in healthcare workers when compared to no intervention [24]. Our study shows that it is likely that hospital personnel would engage in health promotion activities such as receiving tactile massage and hypnosis if the activities were close to the working place; this is also stated in an earlier study [25]. During the health promotion intervention project the participants had the possibility to avail of the health promotion interventions during their shifts. This was much appreciated by the participants because of the high dependency nursing care experienced at the short term emergency ward. Tactile massage therapy as related earlier gave the personnel the ability to continue their shift. The derived categories indicated that it may be valuable to have the possibility in choosing from different types of CTs. Hypnosis or tactile massage may not suit everyone. Hypnosis seemed to be associated with prejudices and may create a fear of losing self-control. However, many of the participants who received hypnosis refer to the increase in their sense of self-control and balance. It seemed that hypnosis provided the participants with an instrument in dealing with self-control both at home and in other places outside the integrative unit. These results are also consistent with other surveys [26] demonstrating the effectiveness of the hypnotic interaction in dealing with negative stress. For the participants who received tactile massage, the therapy seemed to relieve pain. This study reported less physiological pain during the period of health promotion interventions which is also supported in an earlier study [25] where the results indicated a significant reduction in pain intensity and tension levels. There was a significant increase in the degree of relaxation and overall temperament of the personnel. Our findings also indicate that tactile massage may alleviate psychological stress in nurses; this was also a finding in another survey [27]. Both hypnosis and tactile massage seemed to increase the feeling of well-being and the ability in dealing with stress, the impact of well-being seemed to affect both work and leisure time. The increased well-being led to better nursing care of the patients and better cooperation with their colleagues. This is also mentioned in an earlier study which supports that occupational stress leads to conflicts with others [14]. Another positive affect of contentment was when the participants expressed relaxation during health promotion intervention helping them to perform in a different way towards their patients, relatives and colleagues. The participants became more tolerant and patient. During the health promotion intervention period the participants experienced an increased ability in their aptitude to work and increasingly more energy which gave them the strength for a more active social life outside the work place. According to a previous study [28] nurses reported a difficulty in disconnecting from work during their leisure time, and they experienced tiredness and irritation which affected their colleagues, patients and families. Nurses tend to give a lot of themselves to their patients. During this study the participants had the opportunity to be taken care of and that was a very positive experience for them.

### Study limitations

Focus group discussions are valuable in providing rich descriptions of a complex phenomenon; illuminating the experience of unique and unexpected events [29]. There are several limitations to this study. The small number of participants in the focus groups should be considered. This was due to difficulties recruiting participants' ants to the focus group sessions. Due to organizational problems there was a time span of nine months for the third and fourth focus groups interviews. To try to understand the reasons why some of the personnel did not want to participate in the focus group discussions, a survey was conducted among those still working at the hospital (n = 22) during the time the data was analyzed. Twelve members of the personnel responded. The main reasons for not participating in the focus groups were; lack of time, not receiving an invitation to the focus discussions, it was too personal to discuss their experience amongst a group of colleagues. Consequently, in retrospect, individually conducted interviews might have been a better methodological strategy to facilitate higher personnel participation and to gain deeper understanding of the personnel's reported experiences and perceptions. Previous studies including participation in health promotion models show an equally low response rate of 22-30% in nursing personnel, but in those studies no explanation was given to this fact [30,31]. Additional limitations included difficulties in identifying the participants on tape which resulted in quotations named from each focus group and not from a certain participant or in relation to the specific type of health promotion intervention the participant had received.

## Conclusion

The findings indicate that nursing personnel health promotion that integrates tactile massage or hypnosis may help to support some nursing personnel working in a short term emergency ward to deal with a very stressful work environment. This study confirms earlier research suggesting that nursing personnel experience high levels of work-related stress. Tactile massage and hypnosis may hence be useful complements to other health promotion activities as the health promotion interventions allow the body and mind to rest. It may also contribute to positive aspects in the nursing care and improve the nursing personnel's ability to support their patients and colleagues as their tolerance and patience had reached a higher threshold. The health promotion interventions were appreciated by most of the personnel and it gave them the ability to deal with the high dependency nursing care environment. As previously quoted by the participants, they had the energy to continue their shift and enjoy a better social life in their free time. There is a need for further research entailing additional stakeholder perspectives as well as quantitative investigations of clinical and cost-effectiveness before more generalised conclusions can be made.

## Competing interests

The authors declare that they have no competing interests.

## Authors' contributions

The first author (FA) participated in data analysis, drafted the initial manuscript and provided tactile massage in the nursing personnel health promotion project. However, FA was not involved in the data collection or the transcribing of the interviews. SA and TS participated in data gathering, and drafted the manuscript together with FA. To ensure interpretation and validity the derived codes, sub-categories and categories were scrutinized by FA, SA and CF, individually and together. ENH contributed with her knowledge about hypnosis and GÖ with the setting for the study. TF and GÖ critically read the manuscript TF (head) and TS (co-ordinator) developed the overall project design. All authors read and approved the final version of the article.

## Funding

The project was funded by the Ekhaga Foundation (Ekhagastiftelsen).

## Appendix 1

### Interview guide-focus group discussion

• Relate your experience/perceptions from the health promotion project?

• What importance did the treatments have for you personally?

• Bearing in mind the working environment at the short term emergency department, what kind of health activities do you think should be provided?

• Should complementary treatments be included in the health activities provided at the hospital?

• What do you think is the most important aspect in promoting health?

• Do you think that the therapies you received helped you in any way, if so, why?

• Should the treatments have a financial bearing?

• Any ideas or reflections about health promotion?

• Have you ever used complementary treatments before the project?

• Is there anything more you would like to add?

## Pre-publication history

The pre-publication history for this paper can be accessed here:

http://www.biomedcentral.com/1472-6882/11/83/prepub
